# Supplementary material for: A Critical View of the Application of the APEX Software (Aqueous Photochemistry of Environmentally-Occurring Xenobiotics) to Predict Photoreaction Kinetics in Surface Freshwaters
Source: Molecules. 2019 Dec 18;25(1):9. doi: 10.3390/molecules25010009 (PMC7017383; doi:10.3390/molecules25010009)
Supplement: Supplementary file 1 [file molecules-25-00009-s001.zip › 3.2.4_gcc-4.4.0.zip!/3.2.4_gcc-4.4.0/3.2.4_gcc-4.4.0/doc/octave/html/interpreter/Status-of-Variables.html]

Status of Variables - Untitled


Previous: Persistent Variables,
Up: Variables


---

### 7.3 Status of Variables

When creating simple one-shot programs it can be very convenient to
see which variables are available at the prompt. The function `who`
and its siblings `whos` and `whos_line_format` will show
different information about what is in memory, as the following shows.

```
     str = "A random string";
     who -variables
          -| *** local user variables:
          -|
          -| __nargin__  str
```

— Command: **who**  
— Command: **who** pattern ...  
— Command: **who** option pattern ...  
— Command: **C** = who("pattern", ...)  
> List currently defined variables matching the given patterns. Valid
> pattern syntax is the same as described for the `clear` command.
> If no patterns are supplied, all variables are listed.
> By default, only variables visible in the local scope are displayed.
>
> The following are valid options but may not be combined.
>
> `global`: List variables in the global scope rather than the current scope. `-regexp`: The patterns are considered to be regular expressions when matching the variables to display. The same pattern syntax accepted by the `regexp` function is used. `-file`: The next argument is treated as a filename. All variables found within the specified file are listed. No patterns are accepted when reading variables from a file.
>
> If called as a function, return a cell array of defined variable names
> matching the given patterns.
>
>
>
> **See also:** whos, regexp.

— Command: **whos**  
— Command: **whos** pattern ...  
— Command: **whos** option pattern ...  
— Command: **S** = whos("pattern", ...)  
> Provide detailed information on currently defined variables matching the
> given patterns. Options and pattern syntax are the same as for the
> `who` command. Extended information about each variable is
> summarized in a table with the following default entries.
>
> Attr: Attributes of the listed variable. Possible attributes are: blank: Variable in local scope `g`: Variable with global scope `p`: Persistent variable Name: The name of the variable. Size: The logical size of the variable. A scalar is 1x1, a vector is 1xN or Nx1, a 2-D matrix is MxN. Bytes: The amount of memory currently used to store the variable. Class: The class of the variable. Examples include double, single, char, uint16, cell, and struct.
>
> The table can be customized to display more or less information through
> the function `whos_line_format`.
>
> If `whos` is called as a function, return a struct array of defined
> variable names matching the given patterns. Fields in the structure
> describing each variable are: name, size, bytes, class, global, sparse,
> complex, nesting, persistent.
>
>
>
> **See also:** who, whos\_line\_format.

— Built-in Function: val = **whos\_line\_format** ()  
— Built-in Function: old\_val = **whos\_line\_format** (new\_val)  
> Query or set the format string used by the command `whos`.
>
> A full format string is:
>
> ```
>           %[modifier]<command>[:width[:left-min[:balance]]];
> ```
>
> The following command sequences are available:
>
> `%a`: Prints attributes of variables (g=global, p=persistent, f=formal parameter, a=automatic variable). `%b`: Prints number of bytes occupied by variables. `%c`: Prints class names of variables. `%e`: Prints elements held by variables. `%n`: Prints variable names. `%s`: Prints dimensions of variables. `%t`: Prints type names of variables.
>
> Every command may also have an alignment modifier:
>
> `l`: Left alignment. `r`: Right alignment (default). `c`: Column-aligned (only applicable to command %s).
>
> The `width` parameter is a positive integer specifying the minimum
> number of columns used for printing. No maximum is needed as the field will
> auto-expand as required.
>
> The parameters `left-min` and `balance` are only available when the
> column-aligned modifier is used with the command %s.
> `balance` specifies the column number within the field width which will
> be aligned between entries. Numbering starts from 0 which indicates the
> leftmost column. `left-min` specifies the minimum field width to the
> left of the specified balance column.
>
> The default format is
> `" %a:4; %ln:6; %cs:16:6:1; %rb:12; %lc:-1;\n"`.
>
>
>
> **See also:** whos.

Instead of displaying which variables are in memory, it is possible
to determine if a given variable is available. That way it is possible
to alter the behavior of a program depending on the existence of a
variable. The following example illustrates this.

```
     if (! exist ("meaning", "var"))
       disp ("The program has no 'meaning'");
     endif
```

— Built-in Function: **exist** (name, type)  
> Return 1 if the name exists as a variable, 2 if the name is an
> absolute file name, an ordinary file in Octave's `path`, or (after
> appending .m) a function file in Octave's `path`, 3 if the
> name is a .oct or .mex file in Octave's `path`,
> 5 if the name is a built-in function, 7 if the name is a directory, or 103
> if the name is a function not associated with a file (entered on
> the command line).
>
> Otherwise, return 0.
>
> This function also returns 2 if a regular file called name
> exists in Octave's search path. If you want information about
> other types of files, you should use some combination of the functions
> `file_in_path` and `stat` instead.
>
> If the optional argument type is supplied, check only for
> symbols of the specified type. Valid types are
>
> "var": Check only for variables. "builtin": Check only for built-in functions. "file": Check only for files. "dir": Check only for directories.

Usually Octave will manage the memory, but sometimes it can be practical
to remove variables from memory manually. This is usually needed when
working with large variables that fill a substantial part of the memory.
On a computer that uses the IEEE floating point format, the following
program allocates a matrix that requires around 128 MB memory.

```
     large_matrix = zeros (4000, 4000);
```

Since having this variable in memory might slow down other computations,
it can be necessary to remove it manually from memory. The `clear`
function allows this.

— Command: **clear** [options] pattern ...  
> Delete the names matching the given patterns from the symbol table. The
> pattern may contain the following special characters:
>
> `?`: Match any single character. `*`: Match zero or more characters. `[` list `]`: Match the list of characters specified by list. If the first character is `!` or `^`, match all characters except those specified by list. For example, the pattern [a-zA-Z] will match all lower and upper case alphabetic characters.
>
> For example, the command
>
> ```
>           clear foo b*r
> ```
>
> clears the name `foo` and all names that begin with the letter
> `b` and end with the letter `r`.
>
> If `clear` is called without any arguments, all user-defined
> variables (local and global) are cleared from the symbol table. If
> `clear` is called with at least one argument, only the visible
> names matching the arguments are cleared. For example, suppose you have
> defined a function `foo`, and then hidden it by performing the
> assignment `foo = 2`. Executing the command `clear foo` once
> will clear the variable definition and restore the definition of
> `foo` as a function. Executing `clear foo` a second time will
> clear the function definition.
>
> The following options are available in both long and short form
>
> `-all, -a`: Clears all local and global user-defined variables and all functions from the symbol table. `-exclusive, -x`: Clears the variables that don't match the following pattern. `-functions, -f`: Clears the function names and the built-in symbols names. `-global, -g`: Clears the global symbol names. `-variables, -v`: Clears the local variable names. `-classes, -c`: Clears the class structure table and clears all objects. `-regexp, -r`: The arguments are treated as regular expressions as any variables that match will be cleared.
>
> With the exception of `exclusive`, all long options can be used
> without the dash as well.

Information about a function or variable such as its location in the
file system can also be acquired from within Octave. This is usually
only useful during development of programs, and not within a program.

— Command: type **options** name ...  
> Display the definition of each name that refers to a function.
>
> Normally also displays whether each name is user-defined or built-in;
> the `-q` option suppresses this behavior.
>
> If an output argument is requested nothing is displayed. Instead, a cell
> array of strings is returned, where each element corresponds to the
> definition of each requested function.

— Command: **which** name ...  
> Display the type of each name. If name is defined from a
> function file, the full name of the file is also displayed.
>
>
>
> **See also:** help, lookfor.

— Command: **what**  
— Command: **what** dir  
— Function File: w = **what** (dir)  
> List the Octave specific files in a directory. If the variable dir
> is given then check that directory rather than the current directory. If
> a return argument is requested, the files found are returned in the
> structure w.
>
>
>
> **See also:** which.
